# High relative cerebral blood volume is associated with good long term clinical outcomes in acute ischemic stroke: a retrospective cohort study

**DOI:** 10.1186/s12883-024-03806-w

**Published:** 2024-08-26

**Authors:** Marc Strinitz, Claus Zimmer, Maria Berndt, Silke Wunderlich, Tobias Boeckh-Behrens, Christian Maegerlein, Dominik Sepp

**Affiliations:** 1 Department of Diagnostic and Interventional Neuroradiology, Klinikum rechts der Isar, School of Medicine and Health, Technical University of Munich, Munich, Germany; 2 Department of Neurology, Klinikum rechts der Isar, School of Medicine and Health, Technical University of Munich, Munich, Germany

**Keywords:** CT perfusion, Mechanical thrombectomy, RCBV, Ischemic stroke

## Abstract

**Background:**

Endovascular therapy for acute ischemic stroke has been shown to be highly effective in selected patients. However, the ideal criteria for patient selection are still debated. It is well known that collateral flow is an important factor, but the assessment is often subjective and time-consuming. Relative cerebral blood volume (rCBV) is a putative indicator of collateral capacity and can be quickly and easily determined by automated quantitative analysis. We investigated the relationship between rCBV of the affected region and clinical outcome in patients with acute ischemic stroke after endovascular therapy.

**Methods:**

We conducted a retrospective study on consecutive patients between January 2017 and May 2019. Patients with acute ischemic stroke of the anterior circulation who underwent imaging including computed tomography perfusion and were treated with mechanical thrombectomy (MT) were eligible for inclusion. rCBV was calculated automatically with RAPID software by dividing the average cerebral blood volume (CBV) of the affected region (time-to-maximum (Tmax) > 6 s) by the CBV of the unaffected contralateral side. The primary outcome was determined by the modified Rankin Scale (mRS) after 90 days. Good clinical outcome was defined as mRS ≤ 2. We compared means, performed mono- and multivariate logistical regression and calculated a receiver operating characteristic (ROC)-analysis to determine the ideal cutoff value to predict clinical outcomes.

**Results:**

155 patients were enrolled in this study. 66 patients (42.58%) had good clinical outcomes. Higher rCBV was associated with good clinical outcome (*p* < 0.001), even after adjustment for the patients’ status according to mRS and National Institute of Health Stroke Scale (NIHSS) age and Alberta stroke program early computed tomography score (ASPECTS) at baseline (*p* = 0.006). ROC-analysis revealed 0.650 (confidence interval: 0.616–0.778) as the optimal cutoff value.

**Conclusion:**

Higher rCBV at baseline is associated with good clinical long-term outcomes in patients with acute ischemic stroke treated by MT. In this study we provide the biggest collective so far that gives evidence that rCBV can be a valuable tool to identify patients who might benefit from MT and are able give a threshold to help to offer patients MT in borderline cases.

## Background

Acute ischemic stroke is one of the most frequent diseases leading to death or permanent functional deficits in industrialized countries and imposes a high economic burden [1, 2]. Because clinical outcomes are highly dependent on the time window between the initial stroke onset and treatment despite the time of onset being frequently unknown, several methods have been established to identify patients who could benefit from treatment [3].

Final infarct extent can be estimated using the Alberta stroke program early computed tomography score (ASPECTS) at time of presentation in non-contrast cranial computed tomography (NCCT) when optimized reconstruction algorithms are used, but it remains more challenging than approaches combining NCCT with contrast media based imaging techniques [4–6]. An option based on computed tomography angiography (CTA) is to use visual collateralization scores for which subjective grading must be performed [7, 8]. Another approach is to identify the penumbra and the ischemic core in patients with a large vessel occlusion (LVO) who may benefit from mechanical thrombectomy (MT) using CT perfusion (CTP) [9–19]. CTP can identify acutely hypoperfused regions that appear normal on NCCT, and it is therefore recommended in current guidelines for patients with a prolonged time window or unknown time of symptom onset [20, 21]. CTP as a diagnostic tool to identify salvable tissue is not limited to MT but can also be used for intravenous recombinant tissue plasminogen activator (rt-PA) and therefore has a pivotal role in deciding further treatment at time of presentation [22, 23].

The applicability of CTP is based on its ability to objectively depict local collateralization, thus predicting infarct growth in late presenting patients [24–27]. Early CTP mismatch volumes depend on the grade of collateralization, exemplified by occlusions of the internal carotid artery being associated with large absolute mismatch volumes [28]. On the other hand, CTP also has a relevant margin of error, possibly due to a lack of discrimination between penumbra and oligemia making it relevant to optimize the use of its parameters [29–34]. Several CTP parameters have been identified that can be used to estimate local collateralization and whether local brain tissue can still be rescued [23, 35–38]. The most relevant to date are cerebral blood volume (CBV), the hypoperfusion intensity ratio (HIR), relative cerebral blood flow (CBF) < 38% of the unaffected contralateral side and relative CBV (rCBV). HIR, defined as a brain volume with a time-to-maximum longer than 10 s over the brain volume with a time-to-maximum longer than 6 s, correlates well with macrovascular occlusions, low blood pressure, deep tissue infarction and functional outcomes [37, 39, 40]. Relative CBF < 38% is a recently discovered indicator of collateral supply, but the data concerning its benefits and limitation is still scarce [37]. CBV is well established and correlates strongly with final infarct volume [15, 23, 24, 41].

rCBV is defined as the average CBV in the time-to-maximum (Tmax) > 6 s region compared to the average CBV in the normal brain, like CBV it strongly correlates with local collateralization and can be measured objectively [23–28, 37, 42]. Although CTP can provide information about of the affected brain parenchyma, the ability to correctly identify patients with a good chance of functional improvement is even more important than the correct prediction of infarct volume. rCBV has been shown to predict functional outcomes in the posterior and anterior circulation in relatively small studies [25–27, 43, 44]. Because the underlying data are still scarce and therefore the viability of this potentially helpful and objective parameter is unclear, we aim to determine whether rCBV can correctly predict functional outcomes according to the modified Rankin Scale (mRS) after 3 months. Additionally we aim to provide a cutoff value that identifies patients potentially benefitting from MT.

## Methods

### Study design

This study is designed as a retrospective cohort study and adheres to the respective checklist guidelines for strengthening the reporting of observational studies in epidemiology (STROBE) [45]. This study was approved by the ethics committee of the Technical University Munich in accordance with local and regional law. Written informed consent was waived by the ethics committee of the Technical University Munich due to the retrospective design**.** Patients with good clinical outcomes were defined as having a score ≤ 2 on the modified Rankin Scale (mRS) after 90 days. The scale ranges from no symptoms (score of 0) to severe disability and death (score of 5 or 6) and a score ≤ 2 means that the patient is still able to look after himself without assistance from others [46, 47]. Patients with poor clinical outcomes were defined as having a mRS score ≥ 3 after 90 days.

### Inclusion criteria

All consecutive patients who were admitted in the university hospital of the Technical University Munich between January 2017 and May 2019 where eligible for study entry if they met all the following inclusion criteria: 1) The patient suffered from acute ischemic stroke. 2) A LVO of the anterior circulation was diagnosed. 3) A baseline CT with CTP was performed. 4) The baseline mRS at presentation in the university hospital was acquired. 5) Treatment was performed via MT including stroke unit care with or without intra-venous thrombolysis (IVT).

### Exclusion criteria

Patients were excluded from this study when they did not respond to mRS follow-up after 90 days or if rCBV could not be calculated. Other missing data points did not lead to an exclusion but the missing of the data point was noted. Whenever possible we tried to attain the missing values retrospectively.

### Clinical data

The following demographic and clinical data were gathered from our electronic medical records: Biological sex, age and the risk factors for stroke hypertension, hyperlipidemia, diabetes, atrial fibrillation and whether the patient was a current smoker or had previously suffered from a stroke or a transient ischemic attack were collected. Additionally, we noted current treatment with anticoagulants, platelet aggregation inhibitors or statins at the time of presentation and whether the time of the stroke’s onset was known. mRS at presentation and initial severity of the neurological deficit as defined by the National Institute of Health Stroke Scale (NIHSS), a 42 point scale testing for 11 neurological deficits were collected by experienced board-certified neurologists [48]. mRS 3 months after the initial presentation was collected by doctors and nurses from the neurology department who were certified to evaluate mRS score. This was done either during a scheduled visit at the university hospital or by phone interview either by speaking directly to the patient or, if that was not possible, to his or her legal representative. After 3 months routine follow-up ended. Furthermore, CRP and leukocytes at time of presentation were gathered from the laboratory reports. We also gathered whether complications arose during or after treatment, additional intravenous thrombolysis was administered, how many passes were performed during MT and which result according to the modified treatment in cerebral ischemia (TICI) score was achieved [49, 50]. All data points were gathered in a fixed manner in order to reduce selection bias.

### Imaging analysis

ASPECTS at baseline, a parameter for initial infarct extension, was scored according to the respective guideline by judging whether the 10 regions of interest (caudate nucleus, putamen, internal capsule, insular cortex and 6 cortical zones in the hypoperfused media territory) where already showing signs of infarction [6]. ASPECTS scoring was performed by board certified neuroradiologists. LVO location was determined based on our CTA at presentation. CTP parameters were calculated using RAPID Software (iSchemaView, Inc., Menlo Park, California). The ischemic territory was identified by the area showing an increased Tmax of more than 6 s. CBV within the ischemic and nonischemic areas were subsequently calculated. In the next step the mean CBV of the affected territory was divided by the mean CBV of the unaffected territory resulting in the rCBV. HIR was calculated as the ratio of brain volume with time-to-maximum > 10 s over brain volume with time-to-maximum > 6 s. Additionally, the volumes of the infarcted core and the penumbra were gathered.

### Statistical analysis

All parametric and nonparametric tests were calculated using SPSS version 29. Quantitative data were tested for normality by Shapiro–Wilk and Kolmogorov–Smirnov tests and the variables were expressed as mean ± standard deviation (SD) or median ± interquartile range (IQR). Chi-square tests were used to find or exclude differences for categorial variables. Groupwide analyses were performed using the Mann–Whitney U tests or t-tests when applicable. The limit for significance was set to 0.05 a priori. We correlated clinical outcomes as measured by mRS after 90 days in a multivariate analysis with backward elimination. All confounders potentially affecting the outcome available at the time of presentation with a *p*-value lower than 0.1 were included and corrected for. The odds ratio (OR) and confidence intervals (CI) were given when applicable. Receiver operating characteristic (ROC)-analysis was performed for the rCBV to provide the cutoff value with the best sensitivity and specificity for good clinical outcomes after 3 months based on mRS.

## Results

### Basic characteristics of the study population

A total of 230 patients were eligible for study entry. 26 patients did not respond to mRS follow-up after 90 days and were excluded. For 49 patients rCBV could not be determined. Altogether, 155 patients could be included. See Table 1 for demographic, treatment, and outcome data for the groups with good (mRS ≤ 2) or poor (mRS ≥ 3) outcome after 3 months.

**Table 1:** Demographic, therapy and outcome related characteristics for patients with good (mRS ≤ 2) or poor (mRS ≥ 3) outcomes after 3 months

**Demographics**	mRS ≤ 2 at 3 months	mRS ≥ 3 at 3 months	*p*-value
n	66	89	
Age, years (SD)	70.6 (12.6)	79.7 (10.1)	< 0.001
Female sex, n (%)	32 (48.5)	52 (58.4)	0.219
**Medical history**			
Hypertension, n (%)	43 (65.2)	69 (77.5)	0.068
Diabetes mellitus, n (%)	13 (19.7)	25 (28.1)	0.223
Hyperlipidemia, n (%)	22 (33.3)	27 (30.3)	0.602
Atrial fibrillation, n (%)	28 (42.2)	50 (56.2)	0.090
Current smoker, n (%)	9 (13.6)	6 (6.7)	0.339
Anamnestic stroke or TIA, n (%)	7 (10.6)	17 (19.1)	0.132
Treatment with anticoagulants, n (%)	9 (13.6)	26 (29.2)	0.020
Treatment with platelet aggregation inhibitors, n (%)	21 (31.8)	24 (27.0)	0.488
Treatment with statins, n (%)	16 (24.2)	24 (27.0)	0.681
**Clinical presentation**			
NIHSS at presentation (IQR)	7 (4–11)	16 (12–19)	< 0.001
mRS at presentation (IQR)	4 (2–4)	5 (4–5)	< 0.001
Baseline ASPECTS (IQR)	9 (7–10)	8 (6–9)	0.02
rCBV (SD)	0.8 (0.1)	0.7 (0.2)	< 0.001
HIR (SD)	0.3 (0.2)	0.4 (0.2)	0.019
Infarcted core (SD)	7.2 (10.5)	32.4 (45.8)	< 0.001
Penumbra (SD)	91.2 (54.7)	144.0 (193.6)	0.002
CRP at presentation (SD)	0.9 (1.8)	2.0 (3.4)	< 0.001
Leukocytes at presentation (SD)	8.9 (0.9)	9.7 (3.2)	0.075
INR (SD)	1.1 (0.3)	1.1 (0.3)	0.104
**Treatment and results**			
Additional IV rt-PA, n (%)	29 (43.9)	28 (31.5)	0.136
Successful recanalization, n (%)	64 (97.0)	74 (83.1)	0.014
Number of passes, n (SD)	1.9 (1.5)	3 (2.6)	0.017
Complications, n (%)	3 (4.5)	7 (7.9)	0.405
In house mortality, n (%)	0 (0)	18 (20.2)	< 0.001

Values are presented as numbers (percentages) for categorial variables and means (± standard deviation, SD) or medians (± interquartile range, IQR) for continuous variables. *NIHSS *National Institutes of Health Stroke Scale; NIHSS higher than 6 associated with a large vessel occlusion are considered to require further treatment, but lower scores can also be a reason for treatment when the functional deficits are considered disabling. *mRS *modified Rankin Scale; For mRS scores ≥ 3 are defined as poor outcome. mRS ≥ 3 is interpreted as not being able to look after oneself without the assistance of others. mRS scores ≤ 2 are interpreted as not having to rely on the help of others though deficits may be present, they are defined as good outcomes. *ASPECTS *Alberta Stroke Program Early CT score; For ASPECTS values above 6 are considered a relevant volume of salvable tissues in large vessel obstructions of the anterior circulation. *rCBV *relative cerebral blood volume, *HIR *hypoperfusion intensity ratio, infarcted core, ml; penumbra, ml; *CRP *C-reactive protein, mg/dl; leukocytes/nl; *INR *international normalized ratio, *IV rt-PA *intravenous recombinant tissue plasminogen activator

The mean age was 75.8 (12.1) year, and 84 (54.2%) were female. Preexisting medical conditions were hypertension in 112 (72.3%), diabetes mellitus in 38 (24.5%), hyperlipidemia in 49 (31.6%), atrial fibrillation in 78 (50.3%) and continued smoking in 15 (9.7%) patients. 24 (15.5%) patients had a history of stroke or a transient ischemic attack. 35 (22.6%) patients were under treatment with anticoagulants, 45 (29.0%) received platelet aggregation inhibitors and 40 (25.8%) were under treatment with statins. Initial INR was 1.10 (± 0.03).

At presentation, the median NIHSS score was 12 (IQR: 12), and the median mRS score was 4 (IQR: 2). The time of symptom onset was unknown in 61 (39.4%) patients. The median baseline ASPECTS was 9 (IQR: 3). The infarcted core measured 21.71 (± 3.04) ml and the penumbra measured 121.38 (± 7.27) ml. rCBV and HIR were 0.72 (± 0.01) and 0.37 (± 0.02) respectively. While mean C-reactive protein (CRP) at presentation was increased to 1.54 (± 0.26) mg/dl, leukocyte counts were normal at 9.4 (± 0.32) per nl. Additional intravenous treatment was administered in 57 (36.8%) patients. Altogether, 137 (88.4%) patients were successfully (mTICI score ≥ 2b) recanalized in a mean of 2.32 (± 0.16) passes. Complications during the interventional treatment occurred in 10 (6.5%) patients. Of those 4 (2.6%) were embolisms in new territories, 2 (1.3%) were larger postinterventional hemorrhages at arterial puncture, 1 (0.6%) was a self-limited subarachnoid bleeding, 1 (0.6%) was a dissection of the distal internal carotid artery, 1 (0.6%) was a sudden cardiac arrest during treatment followed by an unsuccessful resuscitation and 1 (0.6%) was a tearing of the stent retriever resulting in unsuccessful attempts to remove the device. 66 (42.6%) patients had favorable outcomes. 89 (57.4%) patients had poor clinical outcomes and 18 (11.6%) had died during their hospital stay.

### Outcome analysis

The groups of patients with good and poor clinical outcomes showed no significant differences for sex (*p* = 0.219). Medical history of preexisting hypertension (*p* = 0.068), diabetes mellitus (*p* = 0.223), atrial fibrillation (*p* = 0.090), continued smoking (*p* = 0.339) and a history of stroke (*p* = 0.132) was not more prevalent in either group. Leukocytes at presentation were not significantly different (*p* = 0.075). The international normalized ratio (INR) at time of presentation was 1.1 (0.3) for both groups (*p* = 0.104). There was no significant difference between the groups in terms of the administration of additional intravenous rt-PA (*p* = 0.104), with 29 (43.9%) and 28 (31.5%) respectively. Treatment with antiplatelet agents (*p* = 0.488) or statins (*p* = 0.681) had similar prevalences in both groups. There were no significant differences regarding complications (*p* = 0.405).

Worse outcome was associated with older age (*p* < 0.001), current treatment with anticoagulants (*p* = 0.015), higher CRP levels (*p* < 0.001) and a need for more passes until successful recanalization (*p* = 0.017).

**Fig. 1:**
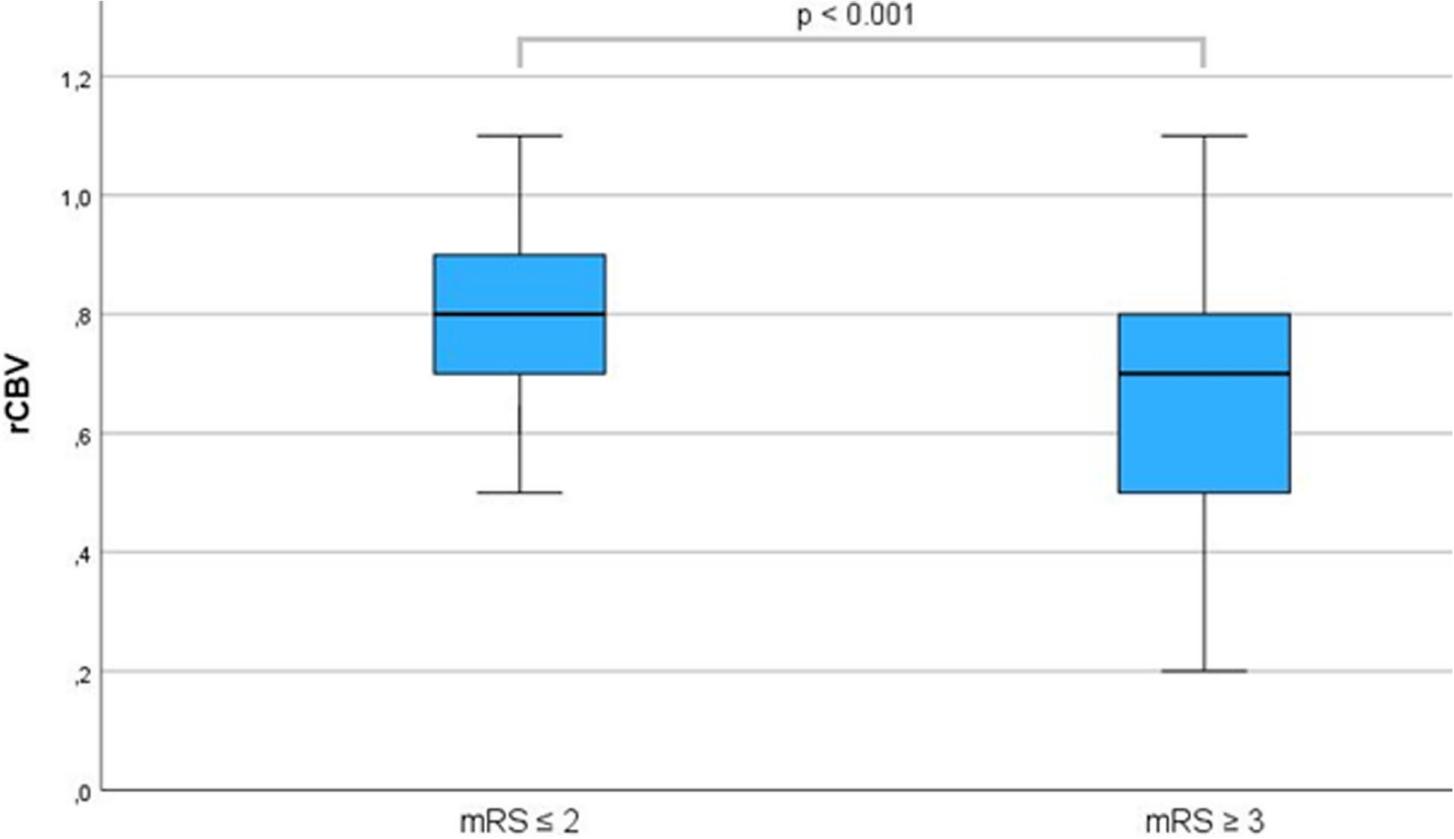
Comparison of boxplots of mean rCBV between patients with mRS ≤ 2 after 90 days and patients with mRS ≥ 3 after 90 days revealed significant differences (*p* < 0.001). For mRS scores ≥ 3 are defined as poor outcome. mRS ≥ 3 is interpreted as not being able to look after oneself without the assistance of others. mRS scores ≤ 2 are interpreted as not having to rely on the help of others though deficits may be present, they are defined as good outcomes. rCBV, relative cerebral blood volume; mRS, modified Rankin Scale

In the group with good outcomes, 64 (97.0%) patients were successfully recanalized while the other group had 74 (83.1%) successful recanalization attempts (*p* < 0.012). Patients with good outcomes were more likely to have a lower NIHSS score at presentation (*p* < 0.001), a lower mRS score at presentation (*p* < 0.001), a higher ASPECTS (*p* = 0.02), a higher rCBV (*p* < 0.001, see Fig. 1), a smaller infarcted core volume (*p* < 0.001), a smaller penumbra (*p* = 0.002) and a lower HIR (*p* = 0.019).

### Multivariate regression analysis

The multivariate regression analysis adjusted for the potential confounders age, NIHSS, mRS and ASPECTS at time of presentation revealed that higher rCBV was associated with a higher likelihood of a favorable outcome, as indicated by a lower mRS score after 90 days (OR: 0.212; CI: 0.93–0.481, *p* = 0.006). The calculated odds ratio suggests that a patient with a rCBV of 0.65 and above is almost 5 times more likely to have a good clinical outcome after 3 months than a patient with rCBV below that value.

### ROC-analysis

**Fig. 2:**
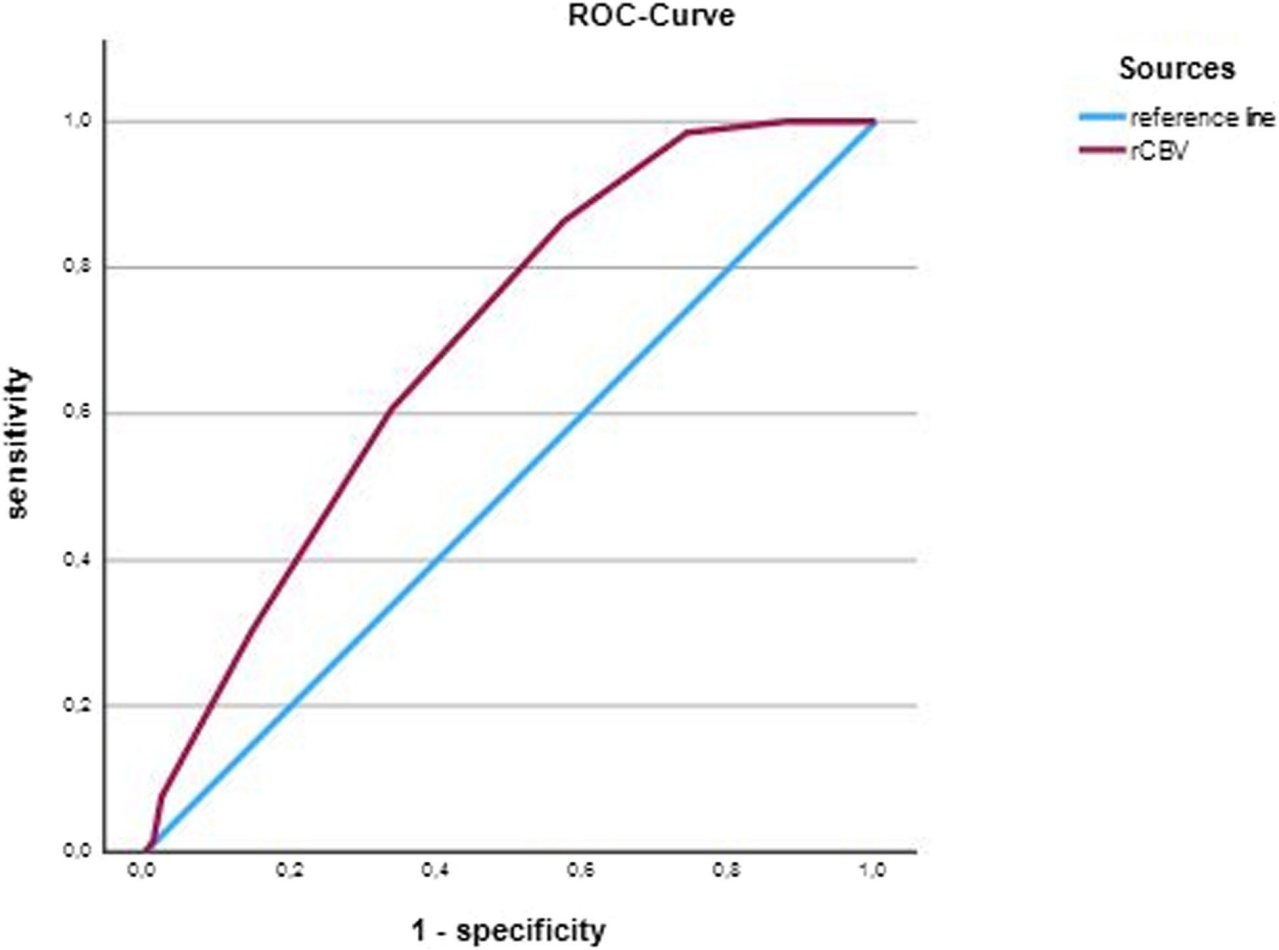
ROC-curve for rCBV values predicting clinical outcomes according to mRS after 3 months. The rCBV at presentation shows a cutoff value of 0.650 (CI: 0.616–0.778) for good clinical outcome after 3 months. AUC: 0.697 (SD: 0.041, *p* < 0.001,). The closer AUC is to 1 the better it functions as a classifier. ROC, receiver operating characteristic; mRS, modified Rankin Scale; rCBV, relative blood volume; CI, confidence interval; SD, standard deviation

The ROC-analysis yielded 0.650 (CI: 0.616–0.778) as the ideal cutoff value for good clinical outcomes after 3 months (Fig. 2). With this threshold a sensitivity of 86.4% and a specificity of 42.7% was calculated. The ROC-analysis found rCBV to predict mRS at 90 days after LVO very well with an area under the curve of 0.697 (SD: 0.041, *p* < 0.001).

## Discussion

In this study we were able to show that rCBV at the time of presentation can identify patients with good clinical outcomes (mRS ≤ 2) after 3 months and we identified a cutoff value of 0.65.

In addition to the main findings, other factors potentially influencing the outcome were analyzed. We found several parameters not derived from the initial CT associated with worse outcome. Similarly to previous studies older age was linked to poor outcomes, likely due to higher prevalence of comorbidities [51]. We also found the previously published association between having suffered a stroke or a transient ischemic attack prior to the current LVO with remaining dependent on help from others [52]. Receiving treatment with anticoagulants before presentation was also associated with worse outcomes this might be because of underlying preexisting vascular diseases. Patients with increased CRP levels at time of presentation were more commonly seen in patients with worse outcomes. This could be due to negative effects due to inflammatory reaction and is in line with similar findings in the past [53]. Worse functional characteristics at baseline measured by mRS and NIHSS were more likely to depend on help by others after 3 months as expected from previous studies [51, 54]. NIHSS of 5 and below is generally considered to be a minor stroke, median NIHSS for both groups were above that threshold with 7 and 16 points respectively as is expected in LVO [48]. We could confirm that patients in whom a higher degree of recanalization according to the mTICI score was achieved with a lower number of passes are more likely to have favorable outcomes after MT [50, 55].

Other parameters seemingly did not influence the outcome after 3 months. Biological sex had no influence in our collective while its overall influence is still debated [56]. We found no impact of medical history concerning previously diagnosed diabetes mellitus, hyperlipidemia or being a smoker. Having a known history of hypertension or atrial fibrillation were not significantly associated with poor outcomes but showed a trend towards worse mRS after 3 months. Neither receiving treatment with platelet aggregation inhibitors nor with statins before the LVO were associated with outcome. We could not show an influence of additional intravenous rt-PA which is potentially caused by the relatively small number of patients, 43% in the group with favorable outcomes and 32% in the group with poor outcomes, that received rt-PA. This falls in line with studies that did not show inferiority of MT alone against a combined treatment approach [57, 58].

All imaging parameters that were included in our study were associated with clinical outcomes. As expected higher ASPECTS in the initial CT scan, which translates to lower volumes of infarcted brain tissue, was linked to favorable outcome [59]. The CTP derived parameters were linked to outcome in the same way. Larger infarcted core volume, identifying tissue that was not salvable even at the time of the initial CTP, was associated with worse outcomes while larger penumbra, meaning the tissue at risk for infarction that could still recover after blood circulation was restored, was linked to favorable outcome as is consistent with current clinical guidelines [20].

The CTP derived parameters that are considered to depict the collateralization found better local perfusion to be associated with better outcomes. HIR, defined as the volume brain with extremely slow time-to-maximum longer than 10 s compared to the brain volume with a time-to-maximum longer than 6 s, showed the expected inverse correlation [37, 39, 40]. While HIR can identify patients who might benefit from MT it is based solely on relative time to maximum. Consequentially, patients with slower cardiac output or a reduced venous outflow capacity might distort this parameter [26].

Other parameters, not applied in this study, that have been used previously to stratify good and poor collateralization and linked them to outcome can be categorized by their respective way of acquisition: The CTP derived parameters CBV, also combining CBV with ASPECTS, and CBF < 30% of the contralateral side are well established and have shown good predictive performance for final infarct volume and clinical outcomes [10, 13, 14, 16, 18, 32, 35, 41, 60]. A relatively new and promising parameter is CBF < 38% of the unaffected contralateral side which performed better than other CTP derived parameters in identifying good collateral flow in a retrospective study but there is still a paucity of data on whether it also is linked to clinical outcomes [37]. CTA-derived collateral scores can be used as indirect measurements for local collateralization. They can be divided between mono- and multiphase CTA. Several monophase CTA scores have been proposed and correlated to clinical outcomes, but they all share the necessity to visually grade the CTA costing some additional time and being at risk of interrater disagreement [61–66]. Multiphase CTA-based scoring is better at predicting final outcomes, but still requires visual grading of the phases while requiring additional scan time and radiation exposure [67, 68]. In the last step before MT is performed angiographic grading systems such as the one proposed by the American Society of Interventional and Therapeutic Neuroradiology/Society of Interventional Radiology (ASITN/SIR) or local time to peak can depict the collateralization, but since they require invasive angiography the decision whether MT is attempted should be made before they can be applied [69–71].

rCBV, which was the main parameter of interest in this retrospective study, showed a strong correlation with outcome even after correction for potential confounders. We identified 0.65 as the ideal cutoff value by ROC-analysis. This is consistent with previously published data that proposed similar cutoff values and found higher rCBV to correlate with favorable outcomes and imaging based surrogate parameters [24–27, 37, 44].

Substantial infarct growth was previously found to be expected with a rCBV of 0.74 and lower while good collateral scores were associated with a rCBV of 0.8 and higher [24, 37]. Additionally, in similar study setups using magnetic resonance imaging-based perfusion at baseline rCBV of 0.8, or 0.85, and higher were associated with good clinical outcome and correlated with blood flow in subsequent digital subtraction angiography [72, 73]. We found that patients with a rCBV of at least 0.65 were almost five times more likely to have a mRS score between 0 and 2 after 3 months, identifying them as very good candidates for MT. Our threshold has a sensitivity of 86.4% and a specificity of 42.7% which means that it is very likely that patients benefitting from MT are likely to be included. The relatively bad specificity can only be tolerated since MT in LVO is a relatively safe treatment [74–77].

This should encourage treating those patients in situations where there has been no clear recommendation for MT. Considering the overarching trend toward attempting MT in borderline cases with patients having lower ASPECTS or lower NIHSS at presentation while being admitted in prolonged intervals between symptom onset and treatment, rCBV could be applied to simplify and objectify the decision regarding MT in those difficult scenarios [9, 78–83]. While in our collective rCBV above a cutoff value of 0.65 was able to predict good clinical outcomes, there is still a lack of data concerning the question whether patients with lower rCBV values may also benefit from MT in a more granular way than mRS after 3 months is able to identify, and consequentially further research is needed limiting the generalizability of this study.

Our study is also limited by its retrospective and monocentric design, making it susceptible to undetected selection bias and an inherent difficulty to control all confounding variables [84]. While we are confident in the overall finding that higher rCBV is associated with good clinical outcome after 3 months a prospective study is needed to prove the applicability of the identified threshold. Another limitation is that 26 patients did not respond to our attempts to determine mRS after 90 days, which could be due to poor outcomes potentially confounding the results. Also, the mRS prior to the current stroke was not acquired systematically which could influence the data by potentially incorrectly associating poor recovery with lower rCBV due to preexisting conditions. In addition, the total group size, albeit larger than in similar studies, is still relatively small which enhances the risk of relatively few data points influencing the overall outcome and potentially changing the results of our ROC-analysis. Another limitation is that perfusion parameters can only depict the small timeframe of during which the scans are performed. Consequently, patients with undulating cerebral perfusion due to arrhythmia or low blood pressure could theoretically influence the results without us having a means to detect them. Thus far this does not seem to be a relevant influence on the CTP.

A strength of the monocentric study design is that all data were acquired within one university hospital therefore we were able to have a highly standardized data acquisition process and homogenous treatment pathways reducing potential bias. Another strength is the relatively large number of patients that could be included in comparison to similar studies which reduces the likelihood of undetected bias. We were able to use the mRS after 90 days as clinical outcome parameter minimizing the influence of short-term effects which can heavily influence the patient's disability in the early post stroke phase [85, 86].

A positive feature of the additional perfusion-derived parameters such as rCBV, especially compared to scores requiring visual grading, is that they can be acquired without causing any additional delays, while also being rater-independent and leading to a clear metric result.

## Conclusions

rCBV can identify patients with favorable treatment outcomes after 90 days at the time of initial presentation in the hospital. Therefore, when established clinical and imaging parameters do not provide a clear recommendation towards mechanical recanalization, rCBV can be a valuable tool to aid in the decision-making process to offer MT in otherwise border line cases.

## Data Availability

Datasets used and/or analyzed during the current study may be obtained from corresponding authors upon reasonable request.

## Abbreviations

ASITN/SIR: American Society of Interventional and Therapeutic Neuroradiology/Society of Interventional Radiology
ASPECTS: Alberta stroke program early CT score
CBV: Cerebral blood volume
CBF: Cerebral blood flow
CI: Confidence interval
CTA: Computed tomography angiography
CTP: Computed tomography perfusion
HIR: Hypoperfusion intensity ratio
LVO: Large vessel occlusion
mRS: Modified Rankin Scale
MT: Mechanical thrombectomy
mTICI score: Modified treatment in cerebral infarction score
NCCT: Non-contrast cranial computed tomography
NIHSS: National Institute of Health Stroke Scale
OR: Odds ratio
rCBV: Relative cerebral blood volume
rt-PA: Recombinant tissue plasminogen activator
ROC: Receiver operating characteristic
STROBE: Strengthening the reporting of observational studies in epidemiology
Tmax: Time-to-maximum
